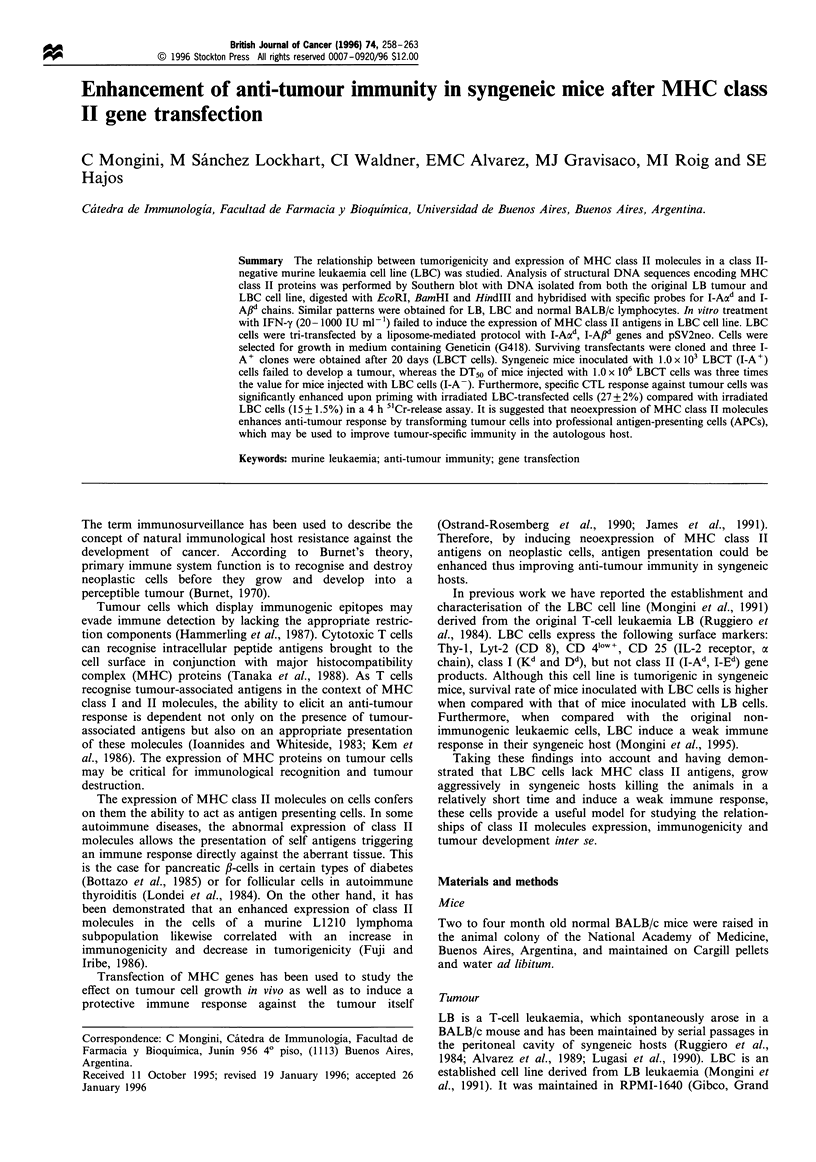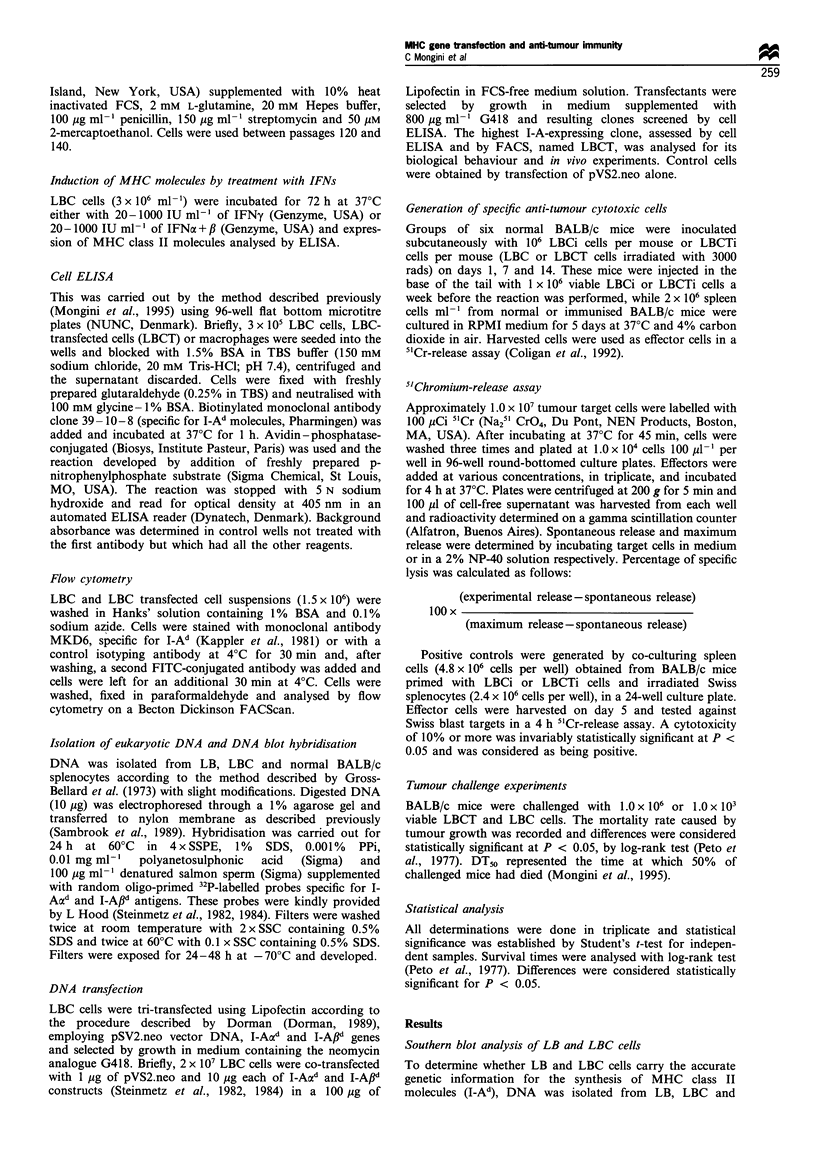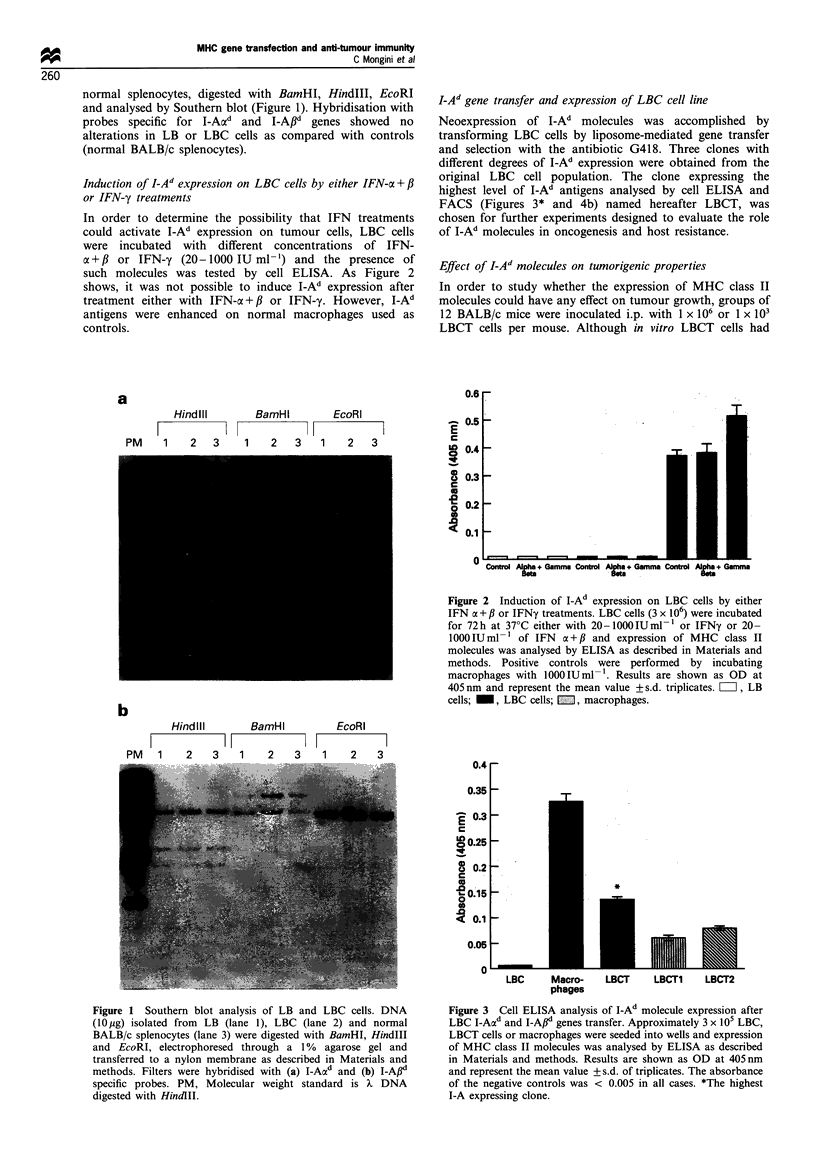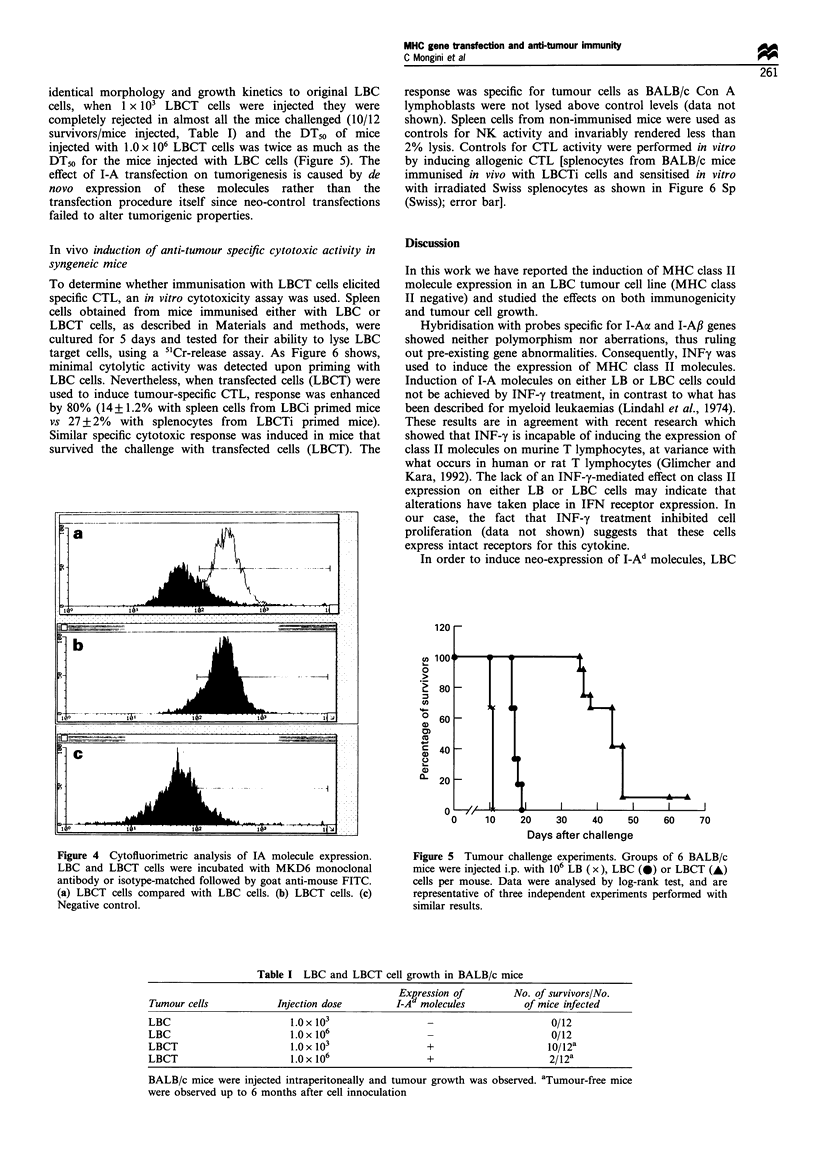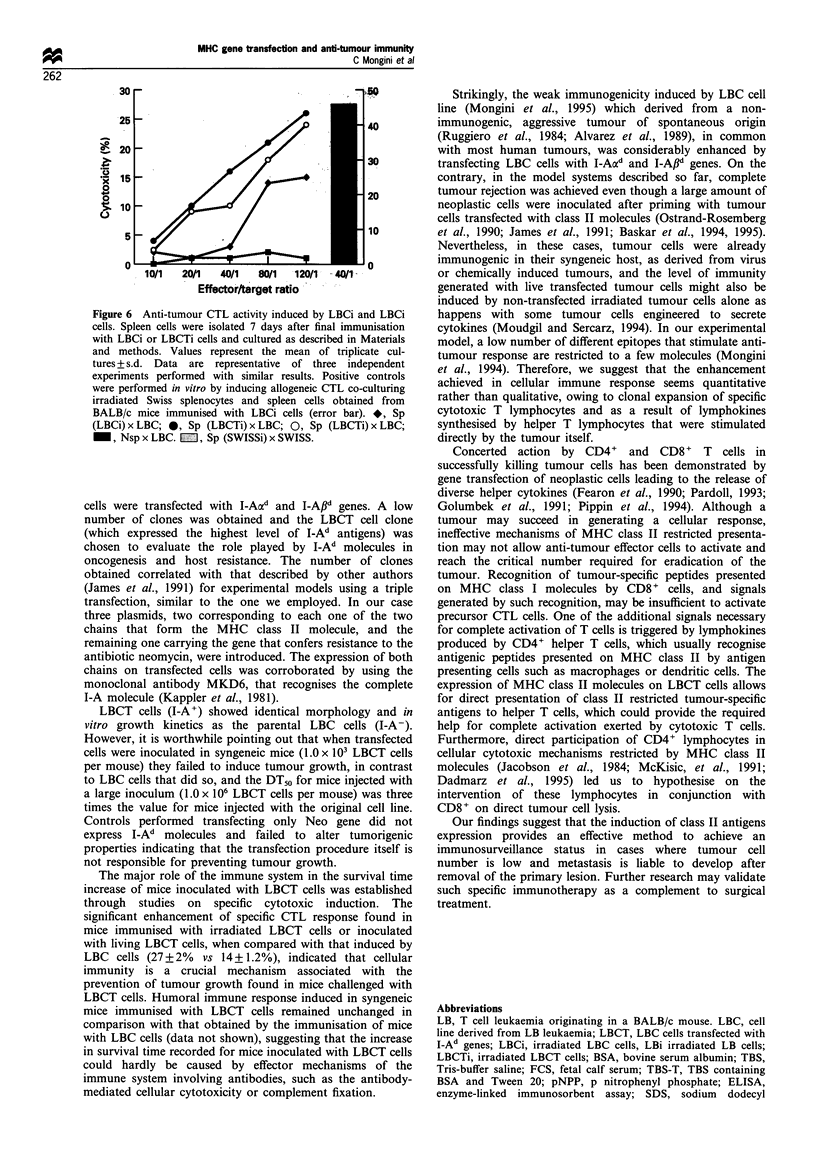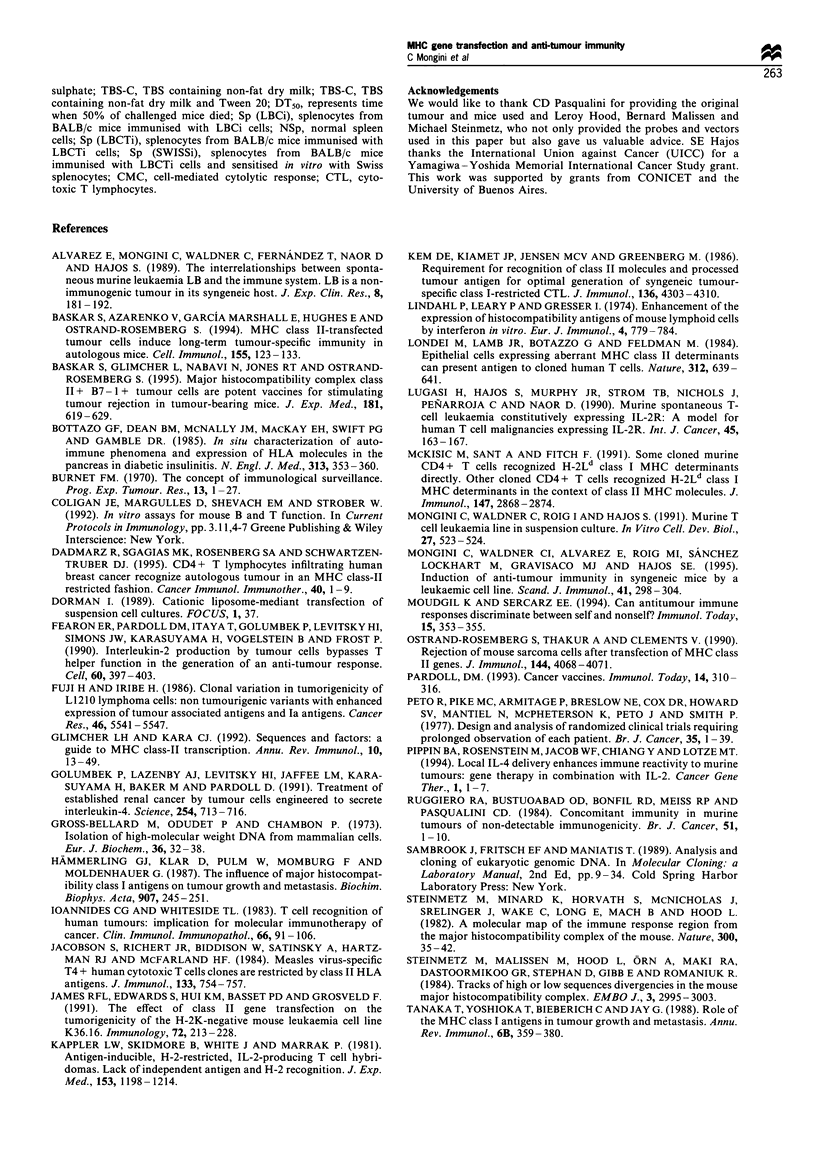# Enhancement of anti-tumour immunity in syngeneic mice after MHC class II gene transfection.

**DOI:** 10.1038/bjc.1996.348

**Published:** 1996-07

**Authors:** C. Mongini, M. Sánchez Lockhart, C. I. Waldner, E. M. Alvarez, M. J. Gravisaco, M. I. Roig, S. E. Hajos

**Affiliations:** Cátedra de Immunología, Facultad de Farmacia y Bioquímica, Universidad de Buenos Aires, Argentina.

## Abstract

**Images:**


British Journal of Cancer (1996) 74, 258-263
© 1996 Stockton Press All rights reserved 0007-0920/96 $12.00

### Enhancement of anti-tumour immunity in syngeneic mice after MHC class II gene transfection

C Mongini, M Sánchez Lockhart, CI Waldner, EMC Alvarez, MJ Gravisaco, MI Roig and SE Hajos

*Cátedra de Immunología, Facultad de Farmacia y Bioquímica, Universidad de Buenos Aires, Buenos Aires, Argentina.*

**Summary** The relationship between tumorigenicity and expression of MHC class II molecules in a class II-negative murine leukaemia cell line (LBC) was studied. Analysis of structural DNA sequences encoding MHC class II proteins was performed by Southern blot with DNA isolated from both the original LB tumour and LBC cell line, digested with *Eco*RI, *Bam*HI and *Hin*dIII and hybridised with specific probes for I-A$\alpha^d$ and I-A$\beta^d$ chains. Similar patterns were obtained for LB, LBC and normal BALB/c lymphocytes. *In vitro* treatment with IFN-$\gamma$ (20-1000 IU ml$^{-1}$) failed to induce the expression of MHC class II antigens in LBC cell line. LBC cells were tri-transfected by a liposome-mediated protocol with I-A$\alpha^d$, I-A$\beta^d$ genes and pSV2neo. Cells were selected for growth in medium containing Geneticin (G418). Surviving transfectants were cloned and three I-A$^+$ clones were obtained after 20 days (LBCT cells). Syngeneic mice inoculated with $1.0 \times 10^3$ LBCT (I-A$^+$) cells failed to develop a tumour, whereas the $DT_{50}$ of mice injected with $1.0 \times 10^6$ LBCT cells was three times the value for mice injected with LBC cells (I-A$^-$). Furthermore, specific CTL response against tumour cells was significantly enhanced upon priming with irradiated LBC-transfected cells ($27 \pm 2\%$) compared with irradiated LBC cells ($15 \pm 1.5\%$) in a 4 h $^{51}$Cr-release assay. It is suggested that neoexpression of MHC class II molecules enhances anti-tumour response by transforming tumour cells into professional antigen-presenting cells (APCs), which may be used to improve tumour-specific immunity in the autologous host.

**Keywords:** murine leukaemia; anti-tumour immunity; gene transfection

The term immunosurveillance has been used to describe the concept of natural immunological host resistance against the development of cancer. According to Burnet's theory, primary immune system function is to recognise and destroy neoplastic cells before they grow and develop into a perceptible tumour (Burnet, 1970).

Tumour cells which display immunogenic epitopes may evade immune detection by lacking the appropriate restriction components (Hammerling *et al.*, 1987). Cytotoxic T cells can recognise intracellular peptide antigens brought to the cell surface in conjunction with major histocompatibility complex (MHC) proteins (Tanaka *et al.*, 1988). As T cells recognise tumour-associated antigens in the context of MHC class I and II molecules, the ability to elicit an anti-tumour response is dependent not only on the presence of tumour-associated antigens but also on an appropriate presentation of these molecules (Ioannides and Whiteside, 1983; Kem *et al.*, 1986). The expression of MHC proteins on tumour cells may be critical for immunological recognition and tumour destruction.

The expression of MHC class II molecules on cells confers on them the ability to act as antigen presenting cells. In some autoimmune diseases, the abnormal expression of class II molecules allows the presentation of self antigens triggering an immune response directly against the aberrant tissue. This is the case for pancreatic $\beta$-cells in certain types of diabetes (Bottazo *et al.*, 1985) or for follicular cells in autoimmune thyroiditis (Londei *et al.*, 1984). On the other hand, it has been demonstrated that an enhanced expression of class II molecules in the cells of a murine L1210 lymphoma subpopulation likewise correlated with an increase in immunogenicity and decrease in tumorigenicity (Fuji and Iribe, 1986).

Transfection of MHC genes has been used to study the effect on tumour cell growth *in vivo* as well as to induce a protective immune response against the tumour itself (Ostrand-Rosemberg *et al.*, 1990; James *et al.*, 1991). Therefore, by inducing neoexpression of MHC class II antigens on neoplastic cells, antigen presentation could be enhanced thus improving anti-tumour immunity in syngeneic hosts.

In previous work we have reported the establishment and characterisation of the LBC cell line (Mongini *et al.*, 1991) derived from the original T-cell leukaemia LB (Ruggiero *et al.*, 1984). LBC cells express the following surface markers: Thy-1, Lyt-2 (CD 8), CD 4$^{low+}$, CD 25 (IL-2 receptor, $\alpha$ chain), class I (K$^d$ and D$^d$), but not class II (I-A$^d$, I-E$^d$) gene products. Although this cell line is tumorigenic in syngeneic mice, survival rate of mice inoculated with LBC cells is higher when compared with that of mice inoculated with LB cells. Furthermore, when compared with the original non-immunogenic leukaemic cells, LBC induce a weak immune response in their syngeneic host (Mongini *et al.*, 1995).

Taking these findings into account and having demonstrated that LBC cells lack MHC class II antigens, grow aggressively in syngeneic hosts killing the animals in a relatively short time and induce a weak immune response, these cells provide a useful model for studying the relationships of class II molecules expression, immunogenicity and tumour development *inter se*.

### Materials and methods

### *Mice*

Two to four month old normal BALB/c mice were raised in the animal colony of the National Academy of Medicine, Buenos Aires, Argentina, and maintained on Cargill pellets and water *ad libitum*.

### *Tumour*

LB is a T-cell leukaemia, which spontaneously arose in a BALB/c mouse and has been maintained by serial passages in the peritoneal cavity of syngeneic hosts (Ruggiero *et al.*, 1984; Alvarez *et al.*, 1989; Lugasi *et al.*, 1990). LBC is an established cell line derived from LB leukaemia (Mongini *et al.*, 1991). It was maintained in RPMI-1640 (Gibco, Grand

Correspondence: C Mongini, Cátedra de Immunología, Facultad de Farmacia y Bioquímica, Junín 956 4° piso, (1113) Buenos Aires, Argentina.
Received 11 October 1995; revised 19 January 1996; accepted 26 January 1996

Island, New York, USA) supplemented with 10% heat inactivated FCS, 2 mM L-glutamine, 20 mM Hepes buffer, 100 $\mu$g ml$^{-1}$ penicillin, 150 $\mu$g ml$^{-1}$ streptomycin and 50 $\mu$M 2-mercaptoethanol. Cells were used between passages 120 and 140.

*Induction of MHC molecules by treatment with IFNs*

LBC cells ($3 \times 10^6$ ml$^{-1}$) were incubated for 72 h at 37°C either with 20–1000 IU ml$^{-1}$ of IFN$\gamma$ (Genzyme, USA) or 20–1000 IU ml$^{-1}$ of IFN$\alpha + \beta$ (Genzyme, USA) and expression of MHC class II molecules analysed by ELISA.

*Cell ELISA*

This was carried out by the method described previously (Mongini *et al.*, 1995) using 96-well flat bottom microtitre plates (NUNC, Denmark). Briefly, $3 \times 10^5$ LBC cells, LBC-transfected cells (LBCT) or macrophages were seeded into the wells and blocked with 1.5% BSA in TBS buffer (150 mM sodium chloride, 20 mM Tris-HCl; pH 7.4), centrifuged and the supernatant discarded. Cells were fixed with freshly prepared glutaraldehyde (0.25% in TBS) and neutralised with 100 mM glycine–1% BSA. Biotinylated monoclonal antibody clone 39–10–8 (specific for I-A$^d$ molecules, Pharmingen) was added and incubated at 37°C for 1 h. Avidin–phosphatase-conjugated (Biosys, Institute Pasteur, Paris) was used and the reaction developed by addition of freshly prepared p-nitrophenylphosphate substrate (Sigma Chemical, St Louis, MO, USA). The reaction was stopped with 5 N sodium hydroxide and read for optical density at 405 nm in an automated ELISA reader (Dynatech, Denmark). Background absorbance was determined in control wells not treated with the first antibody but which had all the other reagents.

*Flow cytometry*

LBC and LBC transfected cell suspensions ($1.5 \times 10^6$) were washed in Hanks' solution containing 1% BSA and 0.1% sodium azide. Cells were stained with monoclonal antibody MKD6, specific for I-A$^d$ (Kappler *et al.*, 1981) or with a control isotyping antibody at 4°C for 30 min and, after washing, a second FITC-conjugated antibody was added and cells were left for an additional 30 min at 4°C. Cells were washed, fixed in paraformaldehyde and analysed by flow cytometry on a Becton Dickinson FACScan.

*Isolation of eukaryotic DNA and DNA blot hybridisation*

DNA was isolated from LB, LBC and normal BALB/c splenocytes according to the method described by Gross-Bellard *et al.* (1973) with slight modifications. Digested DNA (10 $\mu$g) was electrophoresed through a 1% agarose gel and transferred to nylon membrane as described previously (Sambrook *et al.*, 1989). Hybridisation was carried out for 24 h at 60°C in 4×SSPE, 1% SDS, 0.001% PPi, 0.01 mg ml$^{-1}$ polyanetosulphonic acid (Sigma) and 100 $\mu$g ml$^{-1}$ denatured salmon sperm (Sigma) supplemented with random oligo-primed $^{32}$P-labelled probes specific for I-A$\alpha^d$ and I-A$\beta^d$ antigens. These probes were kindly provided by L Hood (Steinmetz *et al.*, 1982, 1984). Filters were washed twice at room temperature with 2×SSC containing 0.5% SDS and twice at 60°C with 0.1×SSC containing 0.5% SDS. Filters were exposed for 24–48 h at −70°C and developed.

*DNA transfection*

LBC cells were tri-transfected using Lipofectin according to the procedure described by Dorman (Dorman, 1989), employing pSV2.neo vector DNA, I-A$\alpha^d$ and I-A$\beta^d$ genes and selected by growth in medium containing the neomycin analogue G418. Briefly, $2 \times 10^7$ LBC cells were co-transfected with 1 $\mu$g of pVS2.neo and 10 $\mu$g each of I-A$\alpha^d$ and I-A$\beta^d$ constructs (Steinmetz *et al.*, 1982, 1984) in a 100 $\mu$g of Lipofectin in FCS-free medium solution. Transfectants were selected by growth in medium supplemented with 800 $\mu$g ml$^{-1}$ G418 and resulting clones screened by cell ELISA. The highest I-A-expressing clone, assessed by cell ELISA and by FACS, named LBCT, was analysed for its biological behaviour and *in vivo* experiments. Control cells were obtained by transfection of pVS2.neo alone.

*Generation of specific anti-tumour cytotoxic cells*

Groups of six normal BALB/c mice were inoculated subcutaneously with $10^6$ LBCi cells per mouse or LBCTi cells per mouse (LBC or LBCT cells irradiated with 3000 rads) on days 1, 7 and 14. These mice were injected in the base of the tail with $1 \times 10^6$ viable LBCi or LBCTi cells a week before the reaction was performed, while $2 \times 10^6$ spleen cells ml$^{-1}$ from normal or immunised BALB/c mice were cultured in RPMI medium for 5 days at 37°C and 4% carbon dioxide in air. Harvested cells were used as effector cells in a $^{51}$Cr-release assay (Coligan *et al.*, 1992).

*$^{51}$Chromium-release assay*

Approximately $1.0 \times 10^7$ tumour target cells were labelled with 100 $\mu$Ci $^{51}$Cr ($Na_2{}^{51}CrO_4$, Du Pont, NEN Products, Boston, MA, USA). After incubating at 37°C for 45 min, cells were washed three times and plated at $1.0 \times 10^4$ cells 100 $\mu$l$^{-1}$ per well in 96-well round-bottomed culture plates. Effectors were added at various concentrations, in triplicate, and incubated for 4 h at 37°C. Plates were centrifuged at 200 ***g*** for 5 min and 100 $\mu$l of cell-free supernatant was harvested from each well and radioactivity determined on a gamma scintillation counter (Alfatron, Buenos Aires). Spontaneous release and maximum release were determined by incubating target cells in medium or in a 2% NP-40 solution respectively. Percentage of specific lysis was calculated as follows:

$$100 \times \frac{\text{(experimental release} - \text{spontaneous release)}}{\text{(maximum release} - \text{spontaneous release)}}$$

Positive controls were generated by co-culturing spleen cells ($4.8 \times 10^6$ cells per well) obtained from BALB/c mice primed with LBCi or LBCTi cells and irradiated Swiss splenocytes ($2.4 \times 10^6$ cells per well), in a 24-well culture plate. Effector cells were harvested on day 5 and tested against Swiss blast targets in a 4 h $^{51}$Cr-release assay. A cytotoxicity of 10% or more was invariably statistically significant at $P < 0.05$ and was considered as being positive.

*Tumour challenge experiments*

BALB/c mice were challenged with $1.0 \times 10^6$ or $1.0 \times 10^3$ viable LBCT and LBC cells. The mortality rate caused by tumour growth was recorded and differences were considered statistically significant at $P < 0.05$, by log-rank test (Peto *et al.*, 1977). $DT_{50}$ represented the time at which 50% of challenged mice had died (Mongini *et al.*, 1995).

*Statistical analysis*

All determinations were done in triplicate and statistical significance was established by Student's *t*-test for independent samples. Survival times were analysed with log-rank test (Peto *et al.*, 1977). Differences were considered statistically significant for $P < 0.05$.

### Results

*Southern blot analysis of LB and LBC cells*

To determine whether LB and LBC cells carry the accurate genetic information for the synthesis of MHC class II molecules (I-A$^d$), DNA was isolated from LB, LBC and normal splenocytes, digested with *Bam*HI, *Hin*dIII, *Eco*RI and analysed by Southern blot (Figure 1). Hybridisation with probes specific for I-A$\alpha^d$ and I-A$\beta^d$ genes showed no alterations in LB or LBC cells as compared with controls (normal BALB/c splenocytes).

*Induction of I-A$^d$ expression on LBC cells by either IFN-$\alpha+\beta$ or IFN-$\gamma$ treatments*

In order to determine the possibility that IFN treatments could activate I-A$^d$ expression on tumour cells, LBC cells were incubated with different concentrations of IFN-$\alpha+\beta$ or IFN-$\gamma$ (20–1000 IU ml$^{-1}$) and the presence of such molecules was tested by cell ELISA. As Figure 2 shows, it was not possible to induce I-A$^d$ expression after treatment either with IFN-$\alpha+\beta$ or IFN-$\gamma$. However, I-A$^d$ antigens were enhanced on normal macrophages used as controls.

*I-A$^d$ gene transfer and expression of LBC cell line*

Neoexpression of I-A$^d$ molecules was accomplished by transforming LBC cells by liposome-mediated gene transfer and selection with the antibiotic G418. Three clones with different degrees of I-A$^d$ expression were obtained from the original LBC cell population. The clone expressing the highest level of I-A$^d$ antigens analysed by cell ELISA and FACS (Figures 3* and 4b) named hereafter LBCT, was chosen for further experiments designed to evaluate the role of I-A$^d$ molecules in oncogenesis and host resistance.

*Effect of I-A$^d$ molecules on tumorigenic properties*

In order to study whether the expression of MHC class II molecules could have any effect on tumour growth, groups of 12 BALB/c mice were inoculated i.p. with $1\times10^6$ or $1\times10^3$ LBCT cells per mouse. Although *in vitro* LBCT cells had

**Figure 1** Southern blot analysis of LB and LBC cells. DNA (10 $\mu$g) isolated from LB (lane 1), LBC (lane 2) and normal BALB/c splenocytes (lane 3) were digested with *Bam*HI, *Hin*dIII and *Eco*RI, electrophoresed through a 1% agarose gel and transferred to a nylon membrane as described in Materials and methods. Filters were hybridised with (**a**) I-A$\alpha^d$ and (**b**) I-A$\beta^d$ specific probes. PM, Molecular weight standard is $\lambda$ DNA digested with *Hind*III.

**Figure 2** Induction of I-A$^d$ expression on LBC cells by either IFN $\alpha+\beta$ or IFN$\gamma$ treatments. LBC cells ($3\times10^6$) were incubated for 72 h at 37°C either with 20–1000 IU ml$^{-1}$ or IFN$\gamma$ or 20–1000 IU ml$^{-1}$ of IFN $\alpha+\beta$ and expression of MHC class II molecules was analysed by ELISA as described in Materials and methods. Positive controls were performed by incubating macrophages with 1000 IU ml$^{-1}$. Results are shown as OD at 405 nm and represent the mean value ±s.d. triplicates. ▭, LB cells; ■, LBC cells; ▒, macrophages.

**Figure 3** Cell ELISA analysis of I-A$^d$ molecule expression after LBC I-A$\alpha^d$ and I-A$\beta^d$ genes transfer. Approximately $3\times10^5$ LBC, LBCT cells or macrophages were seeded into wells and expression of MHC class II molecules was analysed by ELISA as described in Materials and methods. Results are shown as OD at 405 nm and represent the mean value ±s.d. of triplicates. The absorbance of the negative controls was < 0.005 in all cases. *The highest I-A expressing clone.

identical morphology and growth kinetics to original LBC cells, when $1 \times 10^3$ LBCT cells were injected they were completely rejected in almost all the mice challenged (10/12 survivors/mice injected, Table I) and the $DT_{50}$ of mice injected with $1.0 \times 10^6$ LBCT cells was twice as much as the $DT_{50}$ for the mice injected with LBC cells (Figure 5). The effect of I-A transfection on tumorigenesis is caused by *de novo* expression of these molecules rather than the transfection procedure itself since neo-control transfections failed to alter tumorigenic properties.

### In vivo *induction of anti-tumour specific cytotoxic activity in syngeneic mice*

To determine whether immunisation with LBCT cells elicited specific CTL, an *in vitro* cytotoxicity assay was used. Spleen cells obtained from mice immunised either with LBC or LBCT cells, as described in Materials and methods, were cultured for 5 days and tested for their ability to lyse LBC target cells, using a $^{51}$Cr-release assay. As Figure 6 shows, minimal cytolytic activity was detected upon priming with LBC cells. Nevertheless, when transfected cells (LBCT) were used to induce tumour-specific CTL, response was enhanced by 80% ($14 \pm 1.2$% with spleen cells from LBCi primed mice *vs* $27 \pm 2$% with splenocytes from LBCTi primed mice). Similar specific cytotoxic response was induced in mice that survived the challenge with transfected cells (LBCT). The response was specific for tumour cells as BALB/c Con A lymphoblasts were not lysed above control levels (data not shown). Spleen cells from non-immunised mice were used as controls for NK activity and invariably rendered less than 2% lysis. Controls for CTL activity were performed *in vitro* by inducing allogenic CTL [splenocytes from BALB/c mice immunised *in vivo* with LBCTi cells and sensitised *in vitro* with irradiated Swiss splenocytes as shown in Figure 6 Sp (Swiss); error bar].

**Figure 4** Cytofluorimetric analysis of IA molecule expression. LBC and LBCT cells were incubated with MKD6 monoclonal antibody or isotype-matched followed by goat anti-mouse FITC. (**a**) LBCT cells compared with LBC cells. (**b**) LBCT cells. (**c**) Negative control.

### Discussion

In this work we have reported the induction of MHC class II molecule expression in an LBC tumour cell line (MHC class II negative) and studied the effects on both immunogenicity and tumour cell growth.

Hybridisation with probes specific for I-Aα and I-Aβ genes showed neither polymorphism nor aberrations, thus ruling out pre-existing gene abnormalities. Consequently, INFγ was used to induce the expression of MHC class II molecules. Induction of I-A molecules on either LB or LBC cells could not be achieved by INF-γ treatment, in contrast to what has been described for myeloid leukaemias (Lindahl *et al.*, 1974). These results are in agreement with recent research which showed that INF-γ is incapable of inducing the expression of class II molecules on murine T lymphocytes, at variance with what occurs in human or rat T lymphocytes (Glimcher and Kara, 1992). The lack of an INF-γ-mediated effect on class II expression on either LB or LBC cells may indicate that alterations have taken place in IFN receptor expression. In our case, the fact that INF-γ treatment inhibited cell proliferation (data not shown) suggests that these cells express intact receptors for this cytokine.

In order to induce neo-expression of I-A$^d$ molecules, LBC

**Figure 5** Tumour challenge experiments. Groups of 6 BALB/c mice were injected i.p. with $10^6$ LB (×), LBC (●) or LBCT (▲) cells per mouse. Data were analysed by log-rank test, and are representative of three independent experiments performed with similar results.

**Table I** LBC and LBCT cell growth in BALB/c mice

| *Tumour cells* | *Injection dose* | *Expression of I-A$^d$ molecules* | *No. of survivors/No. of mice infected* |
|---|---|---|---|
| LBC | $1.0 \times 10^3$ | – | 0/12 |
| LBC | $1.0 \times 10^6$ | – | 0/12 |
| LBCT | $1.0 \times 10^3$ | + | 10/12[a] |
| LBCT | $1.0 \times 10^6$ | + | 2/12[a] |

BALB/c mice were injected intraperitoneally and tumour growth was observed. [a]Tumour-free mice were observed up to 6 months after cell innoculation

**Figure 6** Anti-tumour CTL activity induced by LBCi and LBCi cells. Spleen cells were isolated 7 days after final immunisation with LBCi or LBCTi cells and cultured as described in Materials and methods. Values represent the mean of triplicate cultures±s.d. Data are representative of three independent experiments performed with similar results. Positive controls were performed *in vitro* by inducing allogeneic CTL co-culturing irradiated Swiss splenocytes and spleen cells obtained from BALB/c mice immunised with LBCi cells (error bar). ◆, Sp (LBCi)×LBC; ●, Sp (LBCTi)×LBC; ○, Sp (LBCTi)×LBC; ■, Nsp×LBC. ▒, Sp (SWISSi)×SWISS.

cells were transfected with I-A$\alpha^d$ and I-A$\beta^d$ genes. A low number of clones was obtained and the LBCT cell clone (which expressed the highest level of I-A$^d$ antigens) was chosen to evaluate the role played by I-A$^d$ molecules in oncogenesis and host resistance. The number of clones obtained correlated with that described by other authors (James *et al.*, 1991) for experimental models using a triple transfection, similar to the one we employed. In our case three plasmids, two corresponding to each one of the two chains that form the MHC class II molecule, and the remaining one carrying the gene that confers resistance to the antibiotic neomycin, were introduced. The expression of both chains on transfected cells was corroborated by using the monoclonal antibody MKD6, that recognises the complete I-A molecule (Kappler *et al.*, 1981).

LBCT cells (I-A$^+$) showed identical morphology and *in vitro* growth kinetics as the parental LBC cells (I-A$^-$). However, it is worthwhile pointing out that when transfected cells were inoculated in syngeneic mice ($1.0\times10^3$ LBCT cells per mouse) they failed to induce tumour growth, in contrast to LBC cells that did so, and the $DT_{50}$ for mice injected with a large inoculum ($1.0\times10^6$ LBCT cells per mouse) was three times the value for mice injected with the original cell line. Controls performed transfecting only Neo gene did not express I-A$^d$ molecules and failed to alter tumorigenic properties indicating that the transfection procedure itself is not responsible for preventing tumour growth.

The major role of the immune system in the survival time increase of mice inoculated with LBCT cells was established through studies on specific cytotoxic induction. The significant enhancement of specific CTL response found in mice immunised with irradiated LBCT cells or inoculated with living LBCT cells, when compared with that induced by LBC cells (27±2% *vs* 14±1.2%), indicated that cellular immunity is a crucial mechanism associated with the prevention of tumour growth found in mice challenged with LBCT cells. Humoral immune response induced in syngeneic mice immunised with LBCT cells remained unchanged in comparison with that obtained by the immunisation of mice with LBC cells (data not shown), suggesting that the increase in survival time recorded for mice inoculated with LBCT cells could hardly be caused by effector mechanisms of the immune system involving antibodies, such as the antibody-mediated cellular cytotoxicity or complement fixation.

Strikingly, the weak immunogenicity induced by LBC cell line (Mongini *et al.*, 1995) which derived from a non-immunogenic, aggressive tumour of spontaneous origin (Ruggiero *et al.*, 1984; Alvarez *et al.*, 1989), in common with most human tumours, was considerably enhanced by transfecting LBC cells with I-A$\alpha^d$ and I-A$\beta^d$ genes. On the contrary, in the model systems described so far, complete tumour rejection was achieved even though a large amount of neoplastic cells were inoculated after priming with tumour cells transfected with class II molecules (Ostrand-Rosemberg *et al.*, 1990; James *et al.*, 1991; Baskar *et al.*, 1994, 1995). Nevertheless, in these cases, tumour cells were already immunogenic in their syngeneic host, as derived from virus or chemically induced tumours, and the level of immunity generated with live transfected tumour cells might also be induced by non-transfected irradiated tumour cells alone as happens with some tumour cells engineered to secrete cytokines (Moudgil and Sercarz, 1994). In our experimental model, a low number of different epitopes that stimulate anti-tumour response are restricted to a few molecules (Mongini *et al.*, 1994). Therefore, we suggest that the enhancement achieved in cellular immune response seems quantitative rather than qualitative, owing to clonal expansion of specific cytotoxic T lymphocytes and as a result of lymphokines synthesised by helper T lymphocytes that were stimulated directly by the tumour itself.

Concerted action by CD4$^+$ and CD8$^+$ T cells in successfully killing tumour cells has been demonstrated by gene transfection of neoplastic cells leading to the release of diverse helper cytokines (Fearon *et al.*, 1990; Pardoll, 1993; Golumbek *et al.*, 1991; Pippin *et al.*, 1994). Although a tumour may succeed in generating a cellular response, ineffective mechanisms of MHC class II restricted presentation may not allow anti-tumour effector cells to activate and reach the critical number required for eradication of the tumour. Recognition of tumour-specific peptides presented on MHC class I molecules by CD8$^+$ cells, and signals generated by such recognition, may be insufficient to activate precursor CTL cells. One of the additional signals necessary for complete activation of T cells is triggered by lymphokines produced by CD4$^+$ helper T cells, which usually recognise antigenic peptides presented on MHC class II by antigen presenting cells such as macrophages or dendritic cells. The expression of MHC class II molecules on LBCT cells allows for direct presentation of class II restricted tumour-specific antigens to helper T cells, which could provide the required help for complete activation exerted by cytotoxic T cells. Furthermore, direct participation of CD4$^+$ lymphocytes in cellular cytotoxic mechanisms restricted by MHC class II molecules (Jacobson *et al.*, 1984; McKisic, *et al.*, 1991; Dadmarz *et al.*, 1995) led us to hypothesise on the intervention of these lymphocytes in conjunction with CD8$^+$ on direct tumour cell lysis.

Our findings suggest that the induction of class II antigens expression provides an effective method to achieve an immunosurveillance status in cases where tumour cell number is low and metastasis is liable to develop after removal of the primary lesion. Further research may validate such specific immunotherapy as a complement to surgical treatment.

**Abbreviations**

LB, T cell leukaemia originating in a BALB/c mouse. LBC, cell line derived from LB leukaemia; LBCT, LBC cells transfected with I-A$^d$ genes; LBCi, irradiated LBC cells, LBi irradiated LB cells; LBCTi, irradiated LBCT cells; BSA, bovine serum albumin; TBS, Tris-buffer saline; FCS, fetal calf serum; TBS-T, TBS containing BSA and Tween 20; pNPP, p nitrophenyl phosphate; ELISA, enzyme-linked immunosorbent assay; SDS, sodium dodecyl sulphate; TBS-C, TBS containing non-fat dry milk; TBS-C, TBS containing non-fat dry milk and Tween 20; $DT_{50}$, represents time when 50% of challenged mice died; Sp (LBCi), splenocytes from BALB/c mice immunised with LBCi cells; NSp, normal spleen cells; Sp (LBCTi), splenocytes from BALB/c mice immunised with LBCTi cells; Sp (SWISSi), splenocytes from BALB/c mice immunised with LBCTi cells and sensitised *in vitro* with Swiss splenocytes; CMC, cell-mediated cytolytic response; CTL, cytotoxic T lymphocytes.

**Acknowledgements**
We would like to thank CD Pasqualini for providing the original tumour and mice used and Leroy Hood, Bernard Malissen and Michael Steinmetz, who not only provided the probes and vectors used in this paper but also gave us valuable advice. SE Hajos thanks the International Union against Cancer (UICC) for a Yamagiwa–Yoshida Memorial International Cancer Study grant. This work was supported by grants from CONICET and the University of Buenos Aires.

### References

ALVAREZ E, MONGINI C, WALDNER C, FERNÁNDEZ T, NAOR D AND HAJOS S. (1989). The interrelationships between spontaneous murine leukaemia LB and the immune system. LB is a non-immunogenic tumour in its syngeneic host. *J. Exp. Clin. Res.*, **8**, 181–192.

BASKAR S, AZARENKO V, GARCÍA MARSHALL E, HUGHES E AND OSTRAND-ROSEMBERG S. (1994). MHC class II-transfected tumour cells induce long-term tumour-specific immunity in autologous mice. *Cell. Immunol.*, **155**, 123–133.

BASKAR S, GLIMCHER L, NABAVI N, JONES RT AND OSTRAND-ROSEMBERG S. (1995). Major histocompatibility complex class II+ B7–1+ tumour cells are potent vaccines for stimulating tumour rejection in tumour-bearing mice. *J. Exp. Med.*, **181**, 619–629.

BOTTAZO GF, DEAN BM, MCNALLY JM, MACKAY EH, SWIFT PG AND GAMBLE DR. (1985). *In situ* characterization of autoimmune phenomena and expression of HLA molecules in the pancreas in diabetic insulinitis. *N. Engl. J. Med.*, **313**, 353–360.

BURNET FM. (1970). The concept of immunological surveillance. *Prog. Exp. Tumour. Res.*, **13**, 1–27.

COLIGAN JE, MARGULLES D, SHEVACH EM AND STROBER W. (1992). *In vitro* assays for mouse B and T function. In *Current Protocols in Immunology*, pp. 3.11,4-7 Greene Publishing & Wiley Interscience: New York.

DADMARZ R, SGAGIAS MK, ROSENBERG SA AND SCHWARTZENTRUBER DJ. (1995). CD4+ T lymphocytes infiltrating human breast cancer recognize autologous tumour in an MHC class-II restricted fashion. *Cancer Immunol. Immunother.*, **40**, 1–9.

DORMAN I. (1989). Cationic liposome-mediant transfection of suspension cell cultures. *FOCUS*, **1**, 37.

FEARON ER, PARDOLL DM, ITAYA T, GOLUMBEK P, LEVITSKY HI, SIMONS JW, KARASUYAMA H, VOGELSTEIN B AND FROST P. (1990). Interleukin-2 production by tumour cells bypasses T helper function in the generation of an anti-tumour response. *Cell*, **60**, 397–403.

FUJI H AND IRIBE H. (1986). Clonal variation in tumorigenicity of L1210 lymphoma cells: non tumourigenic variants with enhanced expression of tumour associated antigens and Ia antigens. *Cancer Res.*, **46**, 5541–5547.

GLIMCHER LH AND KARA CJ. (1992). Sequences and factors: a guide to MHC class-II transcription. *Annu. Rev. Immunol.*, **10**, 13–49.

GOLUMBEK P, LAZENBY AJ, LEVITSKY HI, JAFFEE LM, KARASUYAMA H, BAKER M AND PARDOLL D. (1991). Treatment of established renal cancer by tumour cells engineered to secrete interleukin-4. *Science*, **254**, 713–716.

GROSS-BELLARD M, ODUDET P AND CHAMBON P. (1973). Isolation of high-molecular weight DNA from mammalian cells. *Eur. J. Biochem.*, **36**, 32–38.

HÄMMERLING GJ, KLAR D, PULM W, MOMBURG F AND MOLDENHAUER G. (1987). The influence of major histocompatibility class I antigens on tumour growth and metastasis. *Biochim. Biophys. Acta*, **907**, 245–251.

IOANNIDES CG AND WHITESIDE TL. (1983). T cell recognition of human tumours: implication for molecular immunotherapy of cancer. *Clin. Immunol. Immunopathol.*, **66**, 91–106.

JACOBSON S, RICHERT JR, BIDDISON W, SATINSKY A, HARTZMAN RJ AND MCFARLAND HF. (1984). Measles virus-specific T4+ human cytotoxic T cells clones are restricted by class II HLA antigens. *J. Immunol.*, **133**, 754–757.

JAMES RFL, EDWARDS S, HUI KM, BASSET PD AND GROSVELD F. (1991). The effect of class II gene transfection on the tumorigenicity of the H-2K-negative mouse leukaemia cell line K36.16. *Immunology*, **72**, 213–228.

KAPPLER LW, SKIDMORE B, WHITE J AND MARRAK P. (1981). Antigen-inducible, H-2-restricted, IL-2-producing T cell hybridomas. Lack of independent antigen and H-2 recognition. *J. Exp. Med.*, **153**, 1198–1214.

KEM DE, KIAMET JP, JENSEN MCV AND GREENBERG M. (1986). Requirement for recognition of class II molecules and processed tumour antigen for optimal generation of syngeneic tumour-specific class I-restricted CTL. *J. Immunol.*, **136**, 4303–4310.

LINDAHL P, LEARY P AND GRESSER I. (1974). Enhancement of the expression of histocompatibility antigens of mouse lymphoid cells by interferon *in vitro*. *Eur. J. Immunol.*, **4**, 779–784.

LONDEI M, LAMB JR, BOTAZZO G AND FELDMAN M. (1984). Epithelial cells expressing aberrant MHC class II determinants can present antigen to cloned human T cells. *Nature*, **312**, 639–641.

LUGASI H, HAJOS S, MURPHY JR, STROM TB, NICHOLS J, PEÑARROJA C AND NAOR D. (1990). Murine spontaneous T-cell leukaemia constitutively expressing IL-2R: A model for human T cell malignancies expressing IL-2R. *Int. J. Cancer*, **45**, 163–167.

MCKISIC M, SANT A AND FITCH F. (1991). Some cloned murine CD4+ T cells recognized H-2L$^{d}$ class I MHC determinants directly. Other cloned CD4+ T cells recognized H-2L$^{d}$ class I MHC determinants in the context of class II MHC molecules. *J. Immunol.*, **147**, 2868–2874.

MONGINI C, WALDNER C, ROIG I AND HAJOS S. (1991). Murine T cell leukaemia line in suspension culture. *In Vitro Cell. Dev. Biol.*, **27**, 523–524.

MONGINI C, WALDNER CI, ALVAREZ E, ROIG MI, SÁNCHEZ LOCKHART M, GRAVISACO MJ AND HAJOS SE. (1995). Induction of anti-tumour immunity in syngeneic mice by a leukaemic cell line. *Scand. J. Immunol.*, **41**, 298–304.

MOUDGIL K AND SERCARZ EE. (1994). Can antitumour immune responses discriminate between self and nonself? *Immunol. Today*, **15**, 353–355.

OSTRAND-ROSEMBERG S, THAKUR A AND CLEMENTS V. (1990). Rejection of mouse sarcoma cells after transfection of MHC class II genes. *J. Immunol.*, **144**, 4068–4071.

PARDOLL, DM. (1993). Cancer vaccines. *Immunol. Today*, **14**, 310–316.

PETO R, PIKE MC, ARMITAGE P, BRESLOW NE, COX DR, HOWARD SV, MANTIEL N, MCPHETERSON K, PETO J AND SMITH P. (1977). Design and analysis of randomized clinical trials requiring prolonged observation of each patient. *Br. J. Cancer*, **35**, 1–39.

PIPPIN BA, ROSENSTEIN M, JACOB WF, CHIANG Y AND LOTZE MT. (1994). Local IL-4 delivery enhances immune reactivity to murine tumours: gene therapy in combination with IL-2. *Cancer Gene Ther.*, **1**, 1–7.

RUGGIERO RA, BUSTUOABAD OD, BONFIL RD, MEISS RP AND PASQUALINI CD. (1984). Concomitant immunity in murine tumours of non-detectable immunogenicity. *Br. J. Cancer*, **51**, 1–10.

SAMBROOK J, FRITSCH EF AND MANIATIS T. (1989). Analysis and cloning of eukaryotic genomic DNA. In *Molecular Cloning: a Laboratory Manual*, 2nd Ed, pp. 9–34. Cold Spring Harbor Laboratory Press: New York.

STEINMETZ M, MINARD K, HORVATH S, MCNICHOLAS J, SRELINGER J, WAKE C, LONG E, MACH B AND HOOD L. (1982). A molecular map of the immune response region from the major histocompatibility complex of the mouse. *Nature*, **300**, 35–42.

STEINMETZ M, MALISSEN M, HOOD L, ÖRN A, MAKI RA, DASTOORMIKOO GR, STEPHAN D, GIBB E AND ROMANIUK R. (1984). Tracks of high or low sequences divergencies in the mouse major histocompatibility complex. *EMBO J.*, **3**, 2995–3003.

TANAKA T, YOSHIOKA T, BIEBERICH C AND JAY G. (1988). Role of the MHC class I antigens in tumour growth and metastasis. *Annu. Rev. Immunol.*, **6B**, 359–380.